# A regional registry ensures clinical guideline adherence and economic sustainability of Alzheimer’s disease treatment: a 12-year population-based study in Catalonia

**DOI:** 10.3389/fphar.2026.1848809

**Published:** 2026-07-22

**Authors:** Miriam Umbria, José Exposito, Montserrat Gasol, Antonio Vallano

**Affiliations:** 1 Rational Medicine Use Division, Medicine Area, Catalan Health Service, Barcelona, Spain; 2 Department of Pharmacology, Therapeutics and Toxicology, Autonomous University of Barcelona, Barcelona, Spain

**Keywords:** Alzheimer’s disease, clinical guidelines, drug utilization, health policy, pharmacoepidemiology, population registry, real-world evidence, sustainability

## Abstract

Population-based registries for Alzheimer’s disease pharmacological treatment are essential tools for monitoring clinical guideline adherence and health system sustainability. This retrospective observational study analyzes 12 years of real-world data from the Catalan Registry of Pharmacological Treatment of Alzheimer’s Disease (RTFMA) between 2012 and 2024. Analyzing a cohort of over 158,000 patients treated with acetylcholinesterase inhibitors or memantine, we found that the treated population increased by 24.5%, reaching 56,874 individuals in 2024. The implementation of a mandatory registration system was associated with high clinical appropriateness, with 81.9% of treatments initiated at recommended moderate stages (GDS 4–5), while only 8.6% began at mild stages. Despite the aging population and the impact of the COVID-19 pandemic—which caused a transient decline in treatment initiations in 2020—the system demonstrated resilience with a full recovery by 2023. Treatment discontinuation was primarily driven by mortality (95% of known reasons), indicating high persistence until end-of-life. From an economic perspective, Alzheimer’s therapies represented a stable 1.9% of the total public pharmaceutical budget in 2023, reflecting effective cost containment through generic drug use. These findings suggest that a centralized, guideline-linked registry optimizes patient selection and maintains the economic viability of Alzheimer’s care, providing a robust framework for the future integration of high-cost disease-modifying therapies.

## 1 Introduction

Alzheimer’s disease (AD) represents the primary cause of dementia worldwide, posing a significant burden on healthcare systems due to its progressive nature and the increasing aging of the population (Prince et al., 2013; Scheltens et al., 2021). Currently, pharmacological management remains focused on symptomatic therapies, specifically acetylcholinesterase inhibitors (AChEIs) and memantine, which aim to preserve cognitive and functional abilities in mild-to-moderate stages (Winblad et al., 2016; Tan et al., 2018). Despite the absence of curative treatments, ensuring that these symptomatic drugs are prescribed according to evidence-based clinical guidelines is crucial for optimizing patient outcomes and maintaining health system sustainability (Wimo et al., 2017).

In many healthcare settings, the real-world use of AD medications often deviates from clinical trial evidence, leading to variability in treatment initiation and persistence (Gauthier and Molinuevo, 2013). In Catalonia, efforts to monitor dementia have historically relied on diverse tools, ranging from population-based epidemiological surveillance mechanisms like the Girona Dementia Registry (ReDeGi) (Garre-Olmo et al., 2009) to more recent administrative and clinical databases. To specifically address the variability in pharmacological management, the Catalan Health Service implemented the Registry of Pharmacological Treatment of Alzheimer’s Disease (RTFMA) in 2011. Unlike voluntary or strictly epidemiological databases, the RTFMA is a mandatory regulatory tool integrated into the electronic prescription system (SIRE). It requires clinicians to provide standardized clinical and functional data—such as Global Deterioration Scale (GDS) and Mini-Mental State Examination (MMSE) scores—as a necessary condition to authorize the public funding of AD medications (Umbria et al., 2025). This centralized framework not only monitors clinical appropriateness but also provides a robust source of real-world evidence (RWE) to evaluate the long-term impact of pharmacological policies.

Recent studies in other European regions have shown fluctuating trends in AD drug utilization, often influenced by changes in reimbursement policies and the emergence of generic formulations (Ju et al., 2021; Ippoliti et al., 2023). Furthermore, the COVID-19 pandemic significantly disrupted the management of chronic neurodegenerative diseases, potentially affecting treatment access and continuity for vulnerable populations (Suárez-González et al., 2020; Canevelli et al., 2021). Evaluating how a structured registry-based system like the RTFMA performs under such pressures is vital for understanding the resilience of dementia care pathways.

This study aims to evaluate the 12-year evolution (2012–2024) of symptomatic AD drug use in Catalonia using data from the RTFMA. By analyzing trends in prevalence, incidence, clinical profile at treatment initiation, and economic impact, we seek to demonstrate how a centralized registry ensures adherence to clinical guidelines and supports the economic viability of the healthcare system. Finally, this long-term analysis provides a necessary baseline and a proven regulatory framework for the future integration of emerging high-cost disease-modifying therapies into public health systems.

## 2 Materials and methods

### 2.1 Study design and data source

We conducted a population-based, retrospective observational study using data from the Registry of Pharmacological Treatment of Alzheimer’s Disease (RTFMA) in Catalonia, Spain, covering the period from January 2012 to December 2024. The RTFMA is a mandatory clinical-administrative registry linked to the public health system’s e-prescription platform (Servei Català de la Salut). It captures all patients within the public healthcare network (SISCAT) prescribed acetylcholinesterase inhibitors (donepezil, rivastigmine, galantamine) or the NMDA-receptor antagonist memantine. Since 2012, the registry has served as an automated validation system for drug dispensing. Clinicians are required to update clinical data at least every 24 months for administrative renewal; however, in standard clinical practice, assessments are typically performed every 6–12 months, and the registry allows for real-time updates whenever a significant clinical change occurs or treatment is modified.

### 2.2 Study population and inclusion criteria

The study included all patients who initiated or were actively receiving symptomatic Alzheimer’s disease (AD) pharmacotherapy through the public health system during the study period. A total of 158,527 patients were identified. Inclusion required a formal diagnosis of “probable” or “possible” AD according to the National Institute on Aging–Alzheimer’s Association (NIA-AA) criteria.

### 2.3 Variables and measurements

The following variables were extracted from the RTFMA database:

- Demographics: Age at treatment initiation and sex.
- Clinical Status: AD diagnosis type (probable vs. possible) and baseline cognitive-functional stage as measured by the Global Deterioration Scale (GDS-FAST) and the Mini-Mental State Examination (MMSE). Clinical severity was assessed using data directly extracted from the registry, including the Global Deterioration Scale (GDS-FAST) and the Mini-Mental State Examination (MMSE) scores, which are mandatory fields recorded by clinicians at the time of treatment initiation and during subsequent follow-up validations.
- Treatment Details: Specific drug prescribed at initiation and at the last recorded dispensing, and the use of monotherapy versus combination therapy.
- Temporal Trends: Annual treated prevalence (total patients exposed per year) and annual treatment incidence (defined as new treatment initiations within the registry). To account for demographic changes during the study period, annual incidence and prevalence rates were also calculated per 100,000 inhabitants, stratified by age group and sex, using official census data from the Statistical Institute of Catalonia (Idescat).
- Discontinuation: Reasons for treatment termination were analyzed, including death (exitus), disease progression, adverse effects, lack of efficacy, and loss to follow-up.

### 2.4 Economic analysis

Medication utilization and cost data were obtained from the Catalan Health Service billing datamart (2013–2024). We calculated the total number of dispensations and the gross annual expenditure, distinguishing between public insurer coverage (CatSalut) and patient co-payments. Costs are reported in Euros (€).

### 2.5 Statistical analysis

Descriptive statistics were used to summarize patient characteristics. Continuous variables (e.g., age, treatment duration) are presented as mean (standard deviation, SD) and median (interquartile range, IQR). Categorical variables are expressed as frequencies and percentages. To account for censoring and varying follow-up times, the Restricted Mean Survival Time (RMST) was calculated to provide an interpretable measure of average treatment duration. Time trends were evaluated annually, with specific attention to the impact of the COVID-19 pandemic on incidence and prevalence. Age-specific and sex-specific rates per 100,000 inhabitants were used to evaluate longitudinal trends, allowing for a descriptive comparison that considers the underlying aging structure of the Catalan population. Statistical comparisons between subgroups were performed using Chi-square tests for categorical data and Student’s t-tests (or Welch’s t-test) for continuous variables. All analyses were performed using standardized statistical software.

### 2.6 Ethical approval

The study was approved by the Clinical Research Ethics Committee (CEIm) of the IDIAP Jordi Gol (reference P18/151). Informed consent was waived as the analysis involved anonymized, routinely collected administrative data from a mandatory regulatory registry.

## 3 Results

### 3.1 Treatment population and longitudinal trends

Between 2012 and 2024, a total of 158,527 patients received at least one Alzheimer’s disease (AD) pharmacological treatment through the Catalan public health system. The number of patients actively treated annually showed a progressive increase of 24.5%, rising from 45,629 in 2012 to 56,874 in 2024. Similarly, the number of patients on treatment at year-end grew steadily from 34,085 to 47,835 over the same period. Annual treatment initiations (incidence) typically ranged between 9,000 and 10,900 patients. However, a significant decline was observed in 2020, with only 7,448 new initiations compared to the 2012–2019 annual average of 9,150 (p < 0.001), coinciding with the peak of the COVID-19 pandemic. This was followed by a robust recovery in subsequent years, reaching 10,935 initiations by 2024.

**FIGURE 1:**
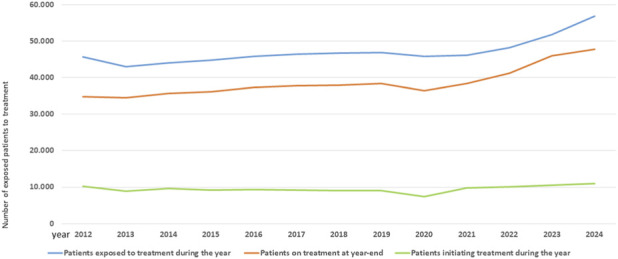
Longitudinal evolution of patients exposed to symptomatic Alzheimer’s disease treatment in Catalonia (2012–2024). The graph illustrates a sustained 24.5% increase in total treated prevalence over the 12-year period. A transient decline in treatment initiations and overall exposure is observed in 2020, corresponding to the impact of the COVID-19 pandemic on healthcare access, followed by a robust recovery to pre-pandemic trends by 2023.

Treatment incidence and prevalence rates were highest among individuals aged 75–84 and ≥85 years. Women consistently showed higher rates than men across all years (p < 0.001, chi-square test), particularly in the oldest age groups. For instance, in the ≥85 age group, the treated prevalence per 100,000 inhabitants was substantially higher in women compared to men, reflecting both a higher disease burden and longer survival (Supplementary Tables S1, S2). Age-specific analysis per 100,000 inhabitants reveals that the growth in treatment utilization is consistent across the older age groups. Between 2012 and 2024, the treated prevalence increased from 5,457.8 to 5,828.5 in the 75–84 age group, and from 4,380.7 to 4,902.5 in those aged ≥85 years (Supplementary Table S2). Furthermore, annual treatment incidence in these segments demonstrated significant resilience; after a decline during the 2020 pandemic peak, initiation rates in the 75–84 and ≥85 groups recovered to 1,134.7 and 1,162.2 per 100,000 inhabitants, respectively, by 2024 (Supplementary Table S1). Discontinuation rates also fluctuated, peaking in 2012 (25.3%) and showing a gradual stabilization toward 2024 (15.9%) (Table 1; Figure 1).

**TABLE 1:** Annual dynamics of Alzheimer’s disease pharmacological treatment in the public health system of Catalonia (2012–2024).

Year	Patients exposed to treatment during the year N (%)	Patients initiating treatment during the year N (%)	Patients with treatment discontinuation during the year N (%)	Patients on treatment at year-end N (%)
2012	45,629 (100)	10,206 (22.4)	11,549 (25.3)	34,085 (74.7)
2013	43,006 (100)	8,921 (20.7)	8,533 (19.8)	34,477 (80.2)
2014	44,100 (100)	9,623 (21.8)	8,453 (19.2)	35,652 (80.8)
2015	44,809 (100)	9,157 (20.4)	8,293 (18.5)	36,517 (81.5)
2016	45,774 (100)	9,257 (20.2)	8,407 (18.4)	37,370 (81.6)
2017	46,499 (100)	9,129 (19.6)	8,728 (18.8)	37,773 (81.2)
2018	46,773 (100)	9,000 (19.2)	8,865 (19.0)	37,909 (81.0)
2019	46,929 (100)	9,020 (19.2)	8,576 (18.3)	38,354 (81.7)
2020	45,802 (100)	7,448 (16.3)	9,335 (20.4)	36,468 (79.6)
2021	46,176 (100)	9,708 (21.0)	7,953 (17.2)	38,224 (82.8)
2022	48,251 (100)	10,027 (20.8)	7,007 (14.5)	41,245 (85.5)
2023	51,817 (100)	10,572 (20.4)	5,878 (11.3)	45,939 (88.7)
2024	56,874 (100)	10,935 (19.2)	9,039 (15.9)	47,835 (84.1)

This table summarizes the yearly treatment patterns among patients exposed to Alzheimer’s disease (AD) medications. “Patients exposed to treatment during the year” refers to all individuals who received at least one prescription of an AD, drug during the calendar year. “Patients initiating treatment during the year” includes those who started AD, therapy in that year without prior exposure. “Patients with treatment discontinuation during the year” refers to individuals whose treatment was permanently discontinued that year. “Patients on treatment at year-end” represents those still receiving AD, treatment as of December 31 of each year.

### 3.2 Patient characteristics and clinical adherence

The demographic and clinical profile of the cohort at treatment initiation is summarized in Table 2. The mean age at initiation was 79.4 years (SD 7.1), with a predominant representation of women (65.4%). Age distribution showed that the vast majority of patients were elderly, with 54.8% aged 75–84 years and 24.2% aged ≥85 years. A diagnosis of probable AD was recorded in 85.8% of cases, while the remaining 13.4% were classified as possible AD, often associated with mixed or vascular components.

**TABLE 2:** Demographic and clinical characteristics of patients at Alzheimer’s disease medication initiation (2012–2024).

Characteristic of patients	Patients (N = 158,517)
**Age** Mean (SD), years Median age (IQR), years	79.4 (7.1) 80 (76–84)
**Age group distribution n (%)** < 65 years 65–74 years 75–84 years ≥85 years	5,302 (3.3) 27,849 (17.6) 86,937 (54.8) 38,439 (24.2)
**Sex, n (%)** Men Women	54,922 (34.6) 103,595 (65.4)
**Diagnosis, n (%)** Probable AD Possible AD* Without information	135,940 (85.8) 21,297 (13.4) 1,290 (0.8)
Cognitive impairment (GDS-FAST), n (%)	
GDS-FAST ≤3 mild cognitive impairment	15,166 (9.6)
GDS-FAST 4 mild dementia	91,820 (57.9)
GDS-FAST 5 moderate dementia	38,108 (24.0)
GDS-FAST 6 moderately severe dementia**	13,402 (8.4)
MMSE score, n (%)	
21–26 mild cognitive impairment	55,084 (34.7)
10–20 moderate cognitive impairment	82,728 (52.2)
<10 severe cognitive impairment	2,878 (1.8)
Other/Without information	17,837 (11.2)

AD: Alzheimer’s disease; GDS-FAST: Global Deterioration Scale-Functional Assessment Staging; MMSE: Mini-Mental State Examination; IQR: interquartile range; SD: Standard Deviation. *Includes mixed dementia, vascular dementia, dementia with Lewy bodies, and Parkinson’s disease dementia. **Aggregated data for stages 6a through 6e.

Regarding clinical appropriateness, the registry documented high adherence to regional guidelines. At the start of therapy, 81.9% of patients were in the recommended stages of dementia, specifically GDS-FAST stage 4 (57.9%) and stage 5 (24.0%). Only a small proportion of patients (8.6%) initiated treatment at the mildest stages (GDS-FAST ≤3), and initiations in very advanced stages (GDS-FAST ≥6) were rare, accounting for less than 10% of the cohort. Baseline MMSE scores further supported this distribution, with 86.9% of patients scoring within the mild (34.7%) or moderate (52.2%) cognitive impairment ranges, demonstrating high internal consistency between different staging tools within the registry.

### 3.3 Drug utilization patterns and treatment duration

Donepezil (38.6%) and rivastigmine (36.5%) were the most frequently prescribed medications at treatment initiation, followed by memantine (10.6%) and galantamine (10.2%) (Table 3). Initial combination therapy (AChEI plus memantine) was utilized in fewer than 4% of cases. Switching patterns were relatively infrequent, with 17.0% of the cohort transitioning to a different monotherapy and 16.5% moving to combination therapy, most commonly from donepezil or galantamine (Supplementary Table S3). Subgroup analysis of the diagnostic categories revealed targeted prescribing patterns. While donepezil was the most frequent choice for “Probable AD” (41.4%), rivastigmine was the predominant monotherapy in subgroups with specific indications, accounting for 38.2% of prescriptions in Dementia with Lewy Bodies and 25.1% in Parkinson’s Disease Dementia. Conversely, rivastigmine use was notably lower in vascular dementia (12.3%). This indicates that, despite the heterogeneous nature of the “possible AD” group, treatment selection aligns with specific clinical profiles (Supplementary Table S4).

**TABLE 3:** Distribution of initial pharmacological treatments for Alzheimer’s disease (2012–2024).

Alzheimer’s drugs	Patients N (%)
Monotherapy	
Donepezil	61,159 (38.6)
Rivastigmine	57,915 (36.5)
Memantine	16,869 (10.6)
Galantamine	16,232 (10.2)
Tacrine	54 (<0.01)
Combination therapy	
Rivastigmine with memantine	2,700 (1.7)
Donepezil with memantine	2,472 (1.6)
Galantamine with memantine	1,126 (0.7)

The mean duration of AD drug treatment was 25.5 months (IQR 8.7–48). The Restricted Mean Survival Time (RMST) for the overall cohort was 41.5 months. Analysis by specific drug showed that galantamine (RMST 46.1 months) and donepezil (RMST 43.3 months) had significantly longer treatment durations compared to memantine (RMST 40.4 months; p < 0.001, Welch’s t-test) (Table 4). Long-term persistence was high, with 7.2% of patients receiving treatment for over 120 months.

**TABLE 4:** Treatment persistence and restricted mean survival time (RMST) for symptomatic Alzheimer’s disease medications (2012–2024).

Medication	RMST (SE) treatment duration (months)*	Median (IQR) duration (months)	Adherence <12 months n (%)	Adherence 12–24 months n (%)	Adherence 24–48 months n (%)**	Adherence ≥48 months n (%)
Donepezil	43.3 (0.13)	27.4 (10.2–51.7)	18,162 (28.3)	10,889 (16.9)	17,096 (26.6)	18,138 (28.2)
Galantamine	46.1 (0.44)	30.3 (11.0–56.4)	4,633 (26.7)	2,651 (15.3)	4,633 (26.7)	5,423 (31.3)
Rivastigmine	41.7 (0.14)	25.2 (9.0–49.3)	19,656 (30.6)	11,424 (17.8)	16,457 (25.7)	16,667 (26.0)
Memantine	40.4 (0.14)	25.3 (10.5–46.8)	17,188 (29.7)	11,269 (19.5)	15,970 (27.6)	13,449 (23.3)
Donepezil + memantine	35.8 (0.22)	21.2 (8.8–40.2)	5,220 (36.3)	3,065 (21.3)	3,668 (25.5)	2,427 (16.8)
Galantamine + memantine	37.1 (0.40)	22.6 (9.2–42.0)	1,395 (31.8)	961 (21.9)	1,182 (26.9)	853 (19.4)
Rivastigmine + memantine	32.6 (0.23)	17.8 (4.0–36.6)	4,812 (42.9)	2,119 (18.9)	2,546 (22.7)	1,730 (15.3)

* RMST, calculated within variable time windows per drug.

** Aggregated data for 24–36 and 36–48 months intervals. SE: standard error; IQR: interquartile range.

### 3.4 Discontinuation and economic impact

At the time of treatment termination, the clinical profile of the cohort reflected advanced disease and older age (Supplementary Table S5). Among the 110,616 patients who discontinued treatment during the study period, the mean age at discontinuation was 83.9 years, with over half of the patients (51.8%) being ≥85 years old. Most patients showed significant cognitive and functional decline before stopping therapy, with 50.7% of those with available staging recorded at GDS-FAST stage 5 or 6, and 53.6% presenting MMSE scores in the moderate-to-severe impairment range (<20). Regarding the reasons for therapy termination, mortality (exitus) was the primary documented driver, accounting for 43.5% of all cases and approximately 95% of cases where a specific clinical reason was provided. Other clinical reasons, such as disease progression (3.5%), adverse events (0.8%), or perceived lack of efficacy (0.5%), were markedly less frequent (Supplementary Table S6). Notably, in half of the discontinuation events (50.0%), the specific reason was not recorded in the registry, often corresponding to passive treatment cessation or unreported deaths.

The economic burden of symptomatic AD medications remained stable relative to the total pharmaceutical budget. Between 2013 and 2024, a total of 4,593,035 dispensations were recorded. While annual dispensations rose by 15.2% (from 357,227 to 411,542), the gross annual expenditure remained relatively constant, ranging from €32.4 million to €37.1 million (Table 5). In 2023, these treatments accounted for approximately 1.9% of the total regional prescription drug spend. The mean cost per dispensation was approximately €94, with 92% of the total cumulative cost (€397 million) covered by the public health system.

**TABLE 5:** Annual outpatient dispensations and associated costs of Alzheimer’s pharmacological treatments (2013–2024)**.** This table summarizes the volume of medications dispensed and the financial burden on the public health system versus patient co-payments.

Year*	Number of dispensations (N)	Gross dispensed amount (€)	Amount covered by the public health system (€)	Patient co-payment amount (€)
2013	357,227	37,100,654.74	35,548,036.75	1,552,608.99
2014	350,340	32,099,478.09	30,558,474.97	1,541,003.12
2015	369,902	33,646,954.69	32,017,266.69	1,629,688.00
2016	372,887	33,939,559.75	32,270,030.16	1,669,529.59
2017	382,457	34,741,539.17	33,022,929.56	1,718,609.61
2018	378,842	33,948,681.42	32,210,379.83	1,738,323.61
2019	382,671	34,075,783.41	32,311,780.13	1,764,003.28
2020	368,424	32,435,652.72	30,664,055.81	1,770,650.91
2021	373,701	32,361,709.10	30,931,324.51	1,430,384.56
2022	388,829	33,523,009.38	32,052,998.08	1,470,010.30
2023	403,910	34,761,254.86	33,140,127.76	1,621,127.10
2024	411,542	35,706,455.83	33,937,369.28	1,769,086.55

* Economic data were not available for 2012.

## 4 Discussion

### 4.1 Principal findings and clinical appropriateness

This study provides a comprehensive population-wide analysis of symptomatic Alzheimer’s disease (AD) medication use in Catalonia over a 12-year period. Our findings indicate that a centralized, mandatory registry is associated with an alignment of clinical practice with evidence-based guidelines. A key highlight is that over 80% of patients initiated treatment at the recommended moderate stages, specifically GDS 4–5. While the lack of a control group limits formal causal inference, the high rate of clinical appropriateness observed (81.9% at GDS 4–5) is intrinsically linked to the registry’s design, which acts as a mandatory gatekeeper for public reimbursement of these therapies. The high internal consistency observed between functional staging (GDS-FAST) and cognitive scores (MMSE) suggests that the risk of ‘coding optimization'—recording stages 4 or 5 solely for reimbursement purposes—is minimal in this registry. This contrasts with other healthcare settings where higher rates of prescribing in mild cognitive impairment or very mild dementia have been observed (Stage et al., 2021). The 9.6% of patients starting therapy at GDS 3 represents those with Mild Cognitive Impairment (MCI). This reflects a specific clinical subgroup where symptomatic treatment is occasionally initiated in real-world practice, despite regional recommendations that prioritize treatment for patients who have already progressed to mild or moderate dementia (GDS 4–5). The negligible use of therapy beyond the severe threshold (GDS ≥7B) indicates that clinicians are successfully adhering to regional protocols which discourage treatment in end-stage dementia (AQuAS, 2016). Furthermore, our findings reveal that pharmacological selection is closely tailored to specific diagnostic etiologies. The preferential use of rivastigmine in patients with Dementia with Lewy Bodies (38.2%) and Parkinson’s Disease Dementia (25.1%) aligns with existing clinical evidence and regulatory indications, which identify rivastigmine as a key therapy for managing cognitive and neuropsychiatric symptoms in these specific subgroups (Wild et al., 2003; Rolinski et al., 2012). This targeted prescribing pattern, contrasted with the lower use of rivastigmine in vascular dementia (12.3%), underscores the registry’s role in reflecting a high degree of diagnostic and therapeutic nuance among clinicians, despite the inherent heterogeneity of the “possible AD” category.

### 4.2 System resilience and the COVID-19 impact

The longitudinal nature of our data captured the significant impact of the COVID-19 pandemic on dementia care. The marked decline in treatment initiations in 2020 reflects the disruption of diagnostic pathways and high mortality among the frail elderly (Ju et al., 2021; Kwon et al., 2024). However, the rapid recovery observed by 2023, with incidence and prevalence rates surpassing pre-pandemic levels, underscores the resilience of the Catalan public health system. This rebound suggests that the registry-based infrastructure (RTFMA) remained a functional tool for monitoring chronic care even under extreme system stress.

### 4.3 International comparison and policy outcomes

Our results help contextualize the “Spanish paradox” noted in recent multinational studies (Reyes et al., 2024). While the United Kingdom saw a doubling of AD drug incidence after NICE guidelines broadened access, Spain reported a decline in new users during the same period (Reyes et al., 2024). Our data clarify that this was not due to a lack of access, but rather to the implementation of the RTFMA and stricter prescribing criteria that curbed initiations in minimally symptomatic cases. Despite this controlled incidence, the overall treated prevalence in Catalonia rose modestly over time, driven by long treatment durations (average of 25.5 months) and the progressive aging of the population. Our age-specific descriptive analysis suggests that the expansion of the registry is driven not only by the absolute increase in the elderly population but also by an increasing rate of treatment initiation within the 75–84 and ≥85 age strata. This indicates that the observed 24.5% increase in the treated population reflects both a growing absolute demand and a broader clinical reach within the increasingly elderly demographic context.

### 4.4 Persistence and the “death as discontinuation” pattern

The finding that mortality (exitus) is the predominant reason for treatment termination is a significant indicator of real-world practice. It implies that once treatment is initiated at the appropriate moderate stage, patients typically remain on therapy until the end of life. However, this interpretation must be framed cautiously given the high rate of unrecorded reasons (50.0%) in our dataset. As non-mortality reasons such as mild adverse events or passive treatment cessation might be less likely to be formally recorded by clinicians, the reported proportion of mortality among documented cases may be overestimated. Even so, the fact that nearly half of all registry exits are due to a documented death underscores the high persistence and the late-stage profile of this treated population. Nevertheless, the robustness of mortality as the primary driver must be weighed against the 50% of cases with missing reasons, which may reflect a reporting bias toward definitive clinical events over more gradual or subjective reasons for cessation. While this highlights high persistence, it also raises questions regarding opportunities for proactive deprescribing in advanced dementia (Reeve et al., 2019; Niznik et al., 2023). Furthermore, treatment dynamics showed that 16.5% of the cohort eventually transitioned to combination therapy, reflecting a clinical response to disease progression and an effort to maintain symptomatic benefits in more advanced stages. As clinicians encounter more patients in the very late stages of the disease, shared decision-making will be critical to weigh modest cognitive benefits against the burdens of polypharmacy and potential adverse effects (Parsons et al., 2021; Alrawiai, 2023).

### 4.5 Economic sustainability and future directions

From a health policy perspective, symptomatic AD treatments have proven to be a sustainable investment, representing only 1.9% of the total regional pharmaceutical budget in 2023. This stability is largely attributable to the widespread availability of generic formulations and the price containment measures introduced in Spain (Vogler et al., 2024; Reyes et al., 2024). It is important to acknowledge that while these external factors reduced the cost per unit, the RTFMA played a critical role in controlling the volume and appropriateness of prescriptions, preventing off-label expansion and ensuring that resources were prioritized for patients within the recommended clinical stages.

Looking ahead, the treatment landscape is shifting with the emergence of disease-modifying therapies, such as monoclonal antibodies targeting amyloid-beta (Cummings et al., 2021; Wu et al., 2023). The high cost and logistical complexity of these new therapies will necessitate strict oversight (Yoon et al., 2024; Ramanan et al., 2023). The Catalan RTFMA provides a ready-made, successful model for managing this transition. By leveraging an existing registry that already ensures clinical appropriateness for symptomatic drugs, health systems can integrate high-cost therapies without abandoning the principles of effective resource allocation.

### 4.6 Strengths and limitations

The primary strength of this study is its population-wide scope, encompassing over 158,000 individuals over a decade of real-world practice. The use of clinical staging (GDS-FAST) at the point of prescription is a unique feature not typically available in administrative databases. This study has several limitations. First, its retrospective and observational nature may introduce inherent biases related to data recording. Additionally, the absence of a geographical or temporal control group (a region without a mandatory registry or a pre-implementation baseline) limits our ability to establish formal causal inferences between the registry’s implementation and the observed clinical outcomes. However, it should be noted that the RTFMA is not a passive observation tool but a mandatory regulatory gatekeeper; the high clinical appropriateness observed (81.9% at GDS 4–5) is directly facilitated by the registry’s design, which requires clinicians to validate disease stages to authorize public reimbursement. Second, the high proportion of missing data regarding discontinuation reasons (50%) is a significant limitation. This missingness may not be at random, potentially leading to an overestimation of mortality as the primary cause of cessation, as clinicians might be more likely to record a definitive event like death than more nuanced reasons like mild adverse effects or shared-decision withdrawal. This data gap necessitates a more conservative interpretation of treatment termination drivers, as the actual impact of non-fatal clinical factors remains partially obscured by administrative missingness. Furthermore, drug exposure was measured through pharmacy dispensing records. While this is a standard and robust proxy for adherence in large population-based studies, it does not guarantee actual ingestion. However, the consistent longitudinal dispensing observed in our cohort strongly suggests high treatment persistence. While the private sector is not captured, the RTFMA includes all patients within the public health system (SISCAT), which covers over 95% of the Catalan population, making these findings highly representative of the regional reality. Regarding the demographic context, while the raw growth in the treated population reflects the region’s shift toward an older population structure, we have addressed this by providing age-specific and sex-specific rates. This descriptive analysis allows for a clearer understanding of treatment reach beyond absolute population volume. Finally, the registry does not systematically capture biomarker data (e.g., CSF or amyloid PET) as part of the diagnostic validation process. Consequently, the diagnosis remains primarily based on clinical and functional criteria, reflecting standard practice in primary and secondary care during the study period.

## 5 Conclusion

The use of symptomatic medications for Alzheimer’s disease in Catalonia from 2012 to 2024 has been characterized by strict adherence to clinical guidelines, consistent with the framework provided by the implementation of a mandatory regional registry. This centralized system supported the observation that the majority of patients initiated therapy at appropriate moderate stages of dementia, optimizing the risk-benefit ratio of these treatments. Despite the transient disruption caused by the COVID-19 pandemic, the healthcare system demonstrated significant resilience, with treatment access and prevalence returning to upward trends by 2023.

Our findings confirm that the Catalan model successfully balances broad access to symptomatic therapies with clinical rigor and economic sustainability, as drug costs remained a small and stable fraction of the total public pharmaceutical budget. Furthermore, the high persistence of treatment until mortality highlights the role of these medications in the long-term management of the disease. As the aging population grows and high-cost disease-modifying therapies emerge, the RTFMA provides a proven and scalable regulatory framework to ensure that future Alzheimer’s care remains effective, equitable, and sustainable.

## Data Availability

The raw data supporting the conclusions of this article will be made available by the authors, without undue reservation.
