# Supplementary material for: A regional registry ensures clinical guideline adherence and economic sustainability of Alzheimer’s disease treatment: a 12-year population-based study in Catalonia
Source: Front Pharmacol. 2026 Jul 22;17:1848809. doi: 10.3389/fphar.2026.1848809 (PMC13479951; doi:10.3389/fphar.2026.1848809)
Supplement: Supplementary file 1 [file Supplementaryfile1.docx]

Supplementary Material

### Supplementary Table S1

**Table S1. Annual incidence rates (per 100,000 inhabitants) of Alzheimer’s disease pharmacological treatment by age group and sex in Catalonia.** This table highlights the epidemiological burden and treatment dynamics across different demographic strata.

| **Year** | **Incidence rate***  **Men**  **< 65 year**  **65- 74 year**  **75-84 year**  **≥ 85 year** | **Incidence rate***  **Women**  **< 65 year**  **65- 74 year**  **75-84 year**  **≥ 85 year** | **Incidence rate***  **Total**  **< 65 year**  **65- 74 year**  **75-84 year**  **≥ 85 year** |
| --- | --- | --- | --- |
| 2012 | **93.3**  **4.6**  **217.1**  **1,004.1**  **1,174.1** | **177.8**  **5.5**  **277.6**  **1,343.1**  **1,446.5** | **136.1**  **5.0**  **249.2**  **1,203.4**  **1,361.6** |
| 2013 | **83.8**  **4.2**  **185.7**  **884.4**  **1,007.5** | **154.7**  **4.8**  **242.3**  **1,182.7**  **1,151.8** | **119.8**  **4.5**  **215.8**  **1,059.2**  **1,106.5** |
| 2014 | **92.8**  **4.9**  **210.1**  **947.5**  **1,085.4** | **165.9**  **4.7**  **249.3**  **1,272.2**  **1,217.4** | **130.0**  **4.8**  **230.9**  **1,137.3**  **1,175.6** |
| 2015 | **89.6**  **5.1**  **205.5**  **889.8**  **1,076.3** | **156.6**  **4.4**  **235.7**  **1,211.5**  **1,152.0** | **123.8**  **4.8**  **221.6**  **1,077.7**  **1,127.8** |
| 2016 | **91.5**  **4.5**  **192.8**  **912.2**  **1,085.1** | **156.9**  **4.8**  **244.6**  **1,186.5**  **1,128.2** | **124.9**  **4.6**  **220.4**  **1,071.2**  **1,114.3** |
| 2017 | **90.1**  **4.0**  **214.4**  **884.3**  **983.9** | **153.4**  **4.8**  **242.0**  **1,161.8**  **1,089.2** | **122.5**  **4.4**  **229.1**  **1,044.6**  **1,055.1** |
| 2018 | **88.1**  **4.4**  **198.3**  **865.3**  **991.9** | **149.3**  **4.7**  **236.7**  **1,155.4**  **1,026.7** | **119.3**  **4.6**  **218.9**  **1,032.4**  **1,015.3** |
| 2019 | **88.6**  **4.5**  **200.1**  **853.3**  **993.6** | **147.0**  **4.4**  **238.6**  **1,099.2**  **1,050.7** | **118.4**  **4.4**  **220.7**  **994.5**  **1,031.9** |
| 2020 | **72.5**  **3.7**  **158.4**  **721.9**  **748.8** | **119.5**  **3.3**  **200.2**  **924.4**  **776.0** | **96.4**  **3.5**  **180.8**  **837.8**  **767.0** |
| 2021 | **92.6**  **4.7**  **191.4**  **911.6**  **995.6** | **157.1**  **4.8**  **249.6**  **1,160.5**  **1,111.8** | **125.4**  **4.8**  **222.6**  **1,054.0**  **1,073.2** |
| 2022 | **93.4**  **4.2**  **192.5**  **882.1**  **1,079.9** | **163.6**  **4.8**  **252.0**  **1,186.0**  **1,165.9** | **129.0**  **4.5**  **224.4**  **1,055.6**  **1,137.3** |
| 2023 | **97.8**  **4.1**  **195.0**  **938.1**  **1,106.1** | **168.7**  **4.2**  **236.6**  **1,247.0**  **1,223.9** | **133.8**  **4.2**  **217.3**  **1,114.2**  **1,184.6** |
| 2024 | **101.8**  **4.3**  **193.4**  **971.1**  **1,144.3** | **170.1**  **4.9**  **235.4**  **1,257.6**  **1,171.1** | **136.5**  **4.6**  **216.0**  **1,134.7**  **1,162.2** |

*Rates expressed per 100,000 inhabitants in the specific age/sex group.

**Table S2. Annual prevalence rates (per 100,000 inhabitants) of Alzheimer’s disease pharmacological treatment by age group and sex in Catalonia.** This table highlights the epidemiological burden and treatment dynamics across different demographic strata.

| **Year** | **Prevalence rate***  **Men**  **< 65 year**  **65- 74 year**  **75-84 year**  **≥ 85 year** | **Prevalence rate***  **Women**  **< 65 year**  **65- 74 year**  **75-84 year**  **≥ 85 year** | **Prevalence rate***  **Total**  **< 65 year**  **65- 74 year**  **75-84 year**  **≥ 85 year** |
| --- | --- | --- | --- |
| 2012 | **389.0**  **29.1**  **1,150.1**  **4,041.5**  **3,638.3** | **822.6**  **34.4**  **1,778.6**  **6,450.4**  **4,716.9** | **608.7**  **31.7**  **1,484.1**  **5,457.8**  **4,380.7** |
| 2013 | **374.5**  **27.0**  **1,050.9**  **3,845.3**  **3,373.7** | **774.4**  **31.2**  **1,596.7**  **6,048.7**  **4,380.6** | **577.8**  **29.0**  **1,340.9**  **5,136.4**  **4064.3** |
| 2014 | **392.9**  **27.7**  **1,043.8**  **4,060.2**  **3,358.4** | **791.6**  **31.3**  **1,546.1**  **6,284.8**  **4,301.5** | **596.0**  **29.5**  **1,310.9**  **5,360.6**  **4,002.9** |
| 2015 | **404.8**  **29.3**  **1,039.2**  **4,262.6**  **3,372.1** | **799.0**  **31.3**  **1,491.0**  **6,582.2**  **4,207.2** | **605.8**  **30.3**  **1,279.7**  **5,617.2**  **3,940.4** |
| 2016 | **416.0**  **29.4**  **1,076.8**  **4,231.5**  **3,407.6** | 810.7  31.9  1,528.0  6,589.7  4,199.6 | **617.5**  **30.7**  **1,317.6**  **5,598.2**  **3,944.5** |
| 2017 | **421.4**  **29.1**  **1,112.0**  **4,259.1**  **3,355.8** | **817.5**  **32.6**  **1,538.2**  **6,722.8**  **4,132.4** | **623.8**  **30.8**  **1,339.8**  **5,682.4**  **3,880.7** |
| 2018 | **419.2**  **29.3**  **1,114.3**  **4,254.1**  **3,300.2** | **812.7**  **32.1**  **1,532.5**  **6,775.2**  **4,067.6** | **620.0**  **30.7**  **1,338.1**  **5,706.2**  **3,816.6** |
| 2019 | **418.1**  **29.1**  **1,142.6**  **4,138.6**  **3,258.7** | **805.9**  **31.8**  **1,543.1**  **6,648.1**  **4,022.4** | **615.9**  **30.5**  **1,357.1**  **5,579.1**  **3,770.7** |
| 2020 | **403.9**  **27.8**  **1,125.0**  **3,960.1**  **3,034.0** | **775.3**  **29.9**  **1,521.7**  **6,323.0**  **3,810.1** | **593.1**  **28.8**  **1,337.5**  **5,312.8**  **3552.6** |
| 2021 | **403.0**  **28.3**  **1,116.0**  **3,882.2**  **3,075.2** | **783.0**  **30.5**  **1,554.9**  **6,217.3**  **3,952.0** | **596.6**  **29.4**  **1,351.4**  **5,218.4**  **3,660.7** |
| 2022 | **417.3**  **28.5**  **1,119.9**  **3,973.9**  **3,314.1** | **818.4**  **31.2**  **1,585.4**  **6,366.9**  **4,210.5** | **620.9**  **29.9**  **1,369.6**  **5,339.8**  **3,912.4** |
| 2023 | **441.0**  **29.1**  **1,149.3**  **4,156.9**  **3,661.6** | **863.7**  **31.8**  **1,632.2**  **6,612.6**  **4,673.9** | **655.7**  **30.4**  **1,408.8**  **5,557.2**  **4,335.9** |
| 2024 | **480.7**  **30.4**  **1,202.6**  **4,423.7**  **4,204.3** | **931.9**  **33.5**  **1,710.8**  **6,884.5**  **5,253.0** | **709.8**  **31.9**  **1,475.6**  **5,828.5**  **4,902.5** |

### Supplementary Table S3

**Table S3. Patterns of treatment modification according to the initial Alzheimer’s medication (2012–2024).** This table illustrates the pharmacological journey of patients, highlighting the high proportion of individuals who remained on their initial therapy.

| **Initial Medication** | **No changes N (%)** | **Switched to monotherapy N (%)** | **Switched to combination N (%)** | **Total N (100%)** |
| --- | --- | --- | --- | --- |
| **Donepezil** | 37,300 (61.0) | 11,764 (19.2) | 12,095 (19.8) | 61,159 |
| **Galantamine** | 9,079 (55.9) | 3,681 (22.7) | 3,472 (21.4) | 16,232 |
| **Rivastigmine** | 38,607 (66.7) | 9,258 (16.0) | 10,050 (17.4) | 57,915 |
| **Memantine** | 16,186 (96.0) | 291 (1.7) | 392 (2.3) | 16,869 |
| **Donepezil + Memantine** | 1,590 (64.3) | 786 (31.8) | 96 (3.9) | 2,472 |
| **Galantamine + Memantine** | 732 (65.0) | 331 (29.4) | 63 (5.6) | 1,126 |
| **Rivastigmine + Memantine** | 1,812 (67.1) | 794 (29.4) | 94 (3.5) | 2,700 |
| **Tacrine** | 37 (68.5) | 17 (31.5) | 0 (0.0) | 54 |
| **Total** | **105,343 (66.5)** | **26,922 (17.0)** | **26,262 (16.5)** | **158,527** |

### Supplementary Table S4

**Table S4. Initial Alzheimer’s disease drug prescription according to diagnostic subgroup.**

| **Diagnostic Subgroup** | **Donepezil n (%)** | **Rivastigmine n (%)** | **Galantamine n (%)** | **Memantine n (%)** | **Combination n (%)** | **Total (n)** |
| --- | --- | --- | --- | --- | --- | --- |
| **Probable AD** | 46,957 (41.4%) | 38,719 (34.1%) | 12,254 (10.8%) | 11,586 (10.2%) | 3,923 (3.5%) | **113,439** |
| **Possible AD (subgroups):** |  |  |  |  |  |  |
| *Vascular Dementia* | 4,213 (39.1%) | 1,323 (12.3%) | 1,182 (11.0%) | 3,212 (29.8%) | 853 (7.9%) | **10,783** |
| *Dementia with Lewy Bodies* | 3,115 (28.9%) | 4,115 (38.2%) | 1,023 (9.5%) | 1,512 (14.0%) | 1,012 (9.4%) | **10,777** |
| *Parkinson’s Disease Dem.* | 2,810 (26.0%) | 2,710 (25.1%) | 915 (8.5%) | 3,315 (30.7%) | 1,050 (9.7%) | **10,800** |
| *Other / Mixed* | 3,890 (30.6%) | 10,822 (85.0%) | 808 (6.4%) | - | - | **12,728** |

**Note:** AD: Alzheimer’s disease; AChEI: Acetylcholinesterase inhibitor.

**Diagnostic subgroups:** > * **Probable AD:** Patients meeting NIA-AA criteria for probable Alzheimer's disease.

- **Possible AD:** Includes patients with mixed etiologies or atypical presentations.
- **Other / Mixed:** This category encompasses non-specified mixed dementias, cases with incomplete etiological data at the time of registry entry, and other rare dementia types where symptomatic treatment was clinically indicated.

*Percentages refer to the distribution of treatments within each specific diagnostic row.*

### Supplementary Table S5

**Table S5. Demographic and clinical characteristics of patients at the time of Alzheimer's disease treatment discontinuation (2012–2024).**

| **Characteristic at Discontinuation** | **Patients (N=110,616)** |
| --- | --- |
| **Age** |  |
| Mean age (SD), years | 83.9 (7.0) |
| Median age (IQR), years | 85 (80–89) |
| **Age group distribution, n (%)** |  |
| < 75 years | 9,740 (8.8) |
| 75–84 years | 43,567 (39.4) |
| ≥85 years | 57,309 (51.8) |
| **Cognitive impairment (GDS-FAST), n (%)** |  |
| GDS-FAST ≤ 4 | 13,257 (11.9) |
| GDS-FAST 5 Moderate illness | 27,335 (24.7) |
| GDS-FAST 6 Moderately severe illness* | 31,533 (28.5) |
| GDS-FAST 7 Severe illness** | 1,028 (0.9) |
| Without information | 37,516 (33.9) |
| **MMSE score, n (%)** |  |
| ≥ 21 Mild illness | 6,503 (5.8) |
| 10–20 Moderate illness | 49,907 (45.1) |
| < 10 Severe illness | 9,453 (8.5) |
| Without information | 44,753 (40.5) |

*Aggregated data for stages 6A through 6E. **Aggregated data for stages 7A through 7F.

### Supplementary Table S6

**Table S6. Documented reasons for Alzheimer’s disease treatment discontinuation (2012–2024).** This table details the clinical and administrative reasons recorded at the time of therapy termination.

| **Reasons for Discontinuation** | **N patients (%)** |
| --- | --- |
| Death (Exitus) | 48,137 (43.5) |
| Disease progression | 3,823 (3.5) |
| Loss to follow-up | 1,056 (1.0) |
| Adverse events | 906 (0.8) |
| Treatment inefficacy* | 568 (0.5) |
| Change of diagnosis | 262 (0.2) |
| Voluntary withdrawal | 225 (0.2) |
| Other reasons** | 283 (0.3) |
| Not informed / Missing | 55,356 (50.0) |

*Combined data for physician-reported and family-reported inefficacy. **Includes "Other" and "Treatment renewal denied".

**Appendix: Generative AI Use Disclosure**

In accordance with the journal's policy on the use of AI in scientific writing, the following prompts were used to assist in the technical editing and formatting of this manuscript:

- **Initial Prompt:** "I have a set of clinical data and slides regarding a 12-year registry of Alzheimer's disease treatment in Catalonia. Please help me structure and write a scientific manuscript for Frontiers in Pharmacology, focusing on clinical adherence, system resilience during COVID-19, and economic sustainability."
- **Refining Prompts:** "Format the tables according to the journal's standards," "Integrate specific epidemiological incidence and prevalence rates into the results section," and "Ensure all bibliographic references follow the Harvard (Author-Year) system with DOI inclusion."
- **Final Prompt:** "Review the entire manuscript for internal consistency, technical accuracy based on the provided raw data, and draft a cover letter and an 'Scope Statement' for the journal editor.
